# Use of proscribed chloroquine is associated with an increased risk of *pfcrt* T76 mutation in some parts of Ghana

**DOI:** 10.1186/1475-2875-13-246

**Published:** 2014-06-26

**Authors:** Kwame K Asare, Johnson N Boampong, Richmond Afoakwah, Elvis O Ameyaw, Rakesh Sehgal, Neils B Quashie

**Affiliations:** 1 Department of Biomedical and Forensic Sciences, University of Cape Coast, Cape Coast, Ghana; 2 Department of Medical Parasitology, Postgraduate Institute of Medical Education and Research, Chandigarh 160012, India; 3 Centre for Tropical Clinical Pharmacology and Therapeutics, University of Ghana Medical School, P O Box GP4236, Accra, Ghana

**Keywords:** *Plasmodium falciparum*, Chloroquine resistant markers, Chloroquine usage, Mutation, *pfcrt*, Ghana

## Abstract

**Background:**

After years of disuse of chloroquine (CQ) as first-line anti-malarial drug in Ghana, reports from molecular studies conducted in parts of the country indicate varying prevalence of T76 mutation in the *pfcrt* gene. This situation has several health implications, one being that mutations that confer resistance to CQ have been reported to show substantial cross-resistance to other anti-malarial drugs. It is important to identify some of the factors contributing to the continuous presence of CQ resistance markers in the country. This study determined the prevalence of T76 mutation in *pfcrt* gene of *Plasmodium falciparum* isolates collected from selected areas of the Central region of Ghana and correlated with the level of CQ use in these areas.

**Methods:**

*Plasmodium falciparum* DNA was extracted from collected blood-blot filter paper samples in the study sites. The prevalence of T76 point mutation in *pfcrt* gene was assessed using nested PCR followed by RFLP. CQ from pharmacy and chemical shops was obtained using mystery buying method. The extent of CQ use by the participants was determined by measuring the level of the drug in their urine samples using the Saker-Solomon method.

**Results:**

Of the 214 *P. falciparum* isolates analysed, 71.9% were found to have T76 mutation of *pfcrt* gene. The study revealed that 14.49% of community pharmacies and chemical shops had stocks of CQ for sale while 16.9% of the participants had CQ in their urine samples. There is five times more risks of becoming infected with CQ resistant strain for staying in an area where CQ is stocked for sale [RR = 0.20, p < 0.0001] and thirteen times more risks of having CQ-resistant mutant from those who still use CQ than non-users [OR = 0.08, p < 0.0001].

**Conclusion:**

This study has shown that high variation in the prevalence of T76 mutations of *P. falciparum* is linked with the level of CQ stocking and usage within study area.

## Background

Undeniably, anti-malarial drug resistance remains a major obstacle to malaria control in the disease endemic areas. Based on reports of high chloroquine (CQ) treatment failure in Ghana and the recommendations of the World Health Organization (WHO) [1-3], CQ was replaced with artemisinin-based combination therapy (ACT) in 2004. Interestingly, high prevalence of CQ resistant mutants have been reported in different parts of the country by several researchers after the change in the malaria treatment policy [4,5] though others have reported decreased trend in prevalence and resistance *in vitro*[6,7].

Substitution of adenylate with cytidylate at position 76 of *Pfcrt* gene which changes lysine to threonine (K76 to T76) is a single molecular marker that strongly correlates with CQ drug resistance in *Plasmodium falciparum* in most malaria endemic areas [8,9]. Similarly, other mutations at different codons of *pfcrt* gene have also shown association with CQ resistance [8,10,11].

In a retrospective study conducted from 2003 to 2010 by Duah and colleagues, they showed that there is decrease in T76, although the prevalence varies from study site to study site [6]. Earlier, Abruquah and colleagues had reported 88.6% K76T prevalence in Kumasi [4], while Kwansa-Bentum and colleagues reported 51.6% K76T prevalence in southern part of Ghana [5]. A study conducted in 2004 by Duah and colleagues reported different T76 mutations among the various sentinel sites for their studies [12]. In that study, they reported T76 prevalence to be 80% at Hohoe, 46% at Navrongo, 98% at Tarkwa, 61% at Sunyani & 46% in Yendi [12]. This study was conducted to find out the prevalence of T76 mutation in the selected health facilities and to determine the cause for the high prevalence of CQ resistance.

## Methods

### Study sites

Samples for this study were collected from municipal hospital (Cape Coast), district hospitals (Twifo Praso and Assin Foso) and health centres (Elmina) all in the Central region of Ghana. Cape Coast metropolis covers an area of 122 square kilometres (sq. km) and is the smallest metropolis in the country. The capital, Cape Coast, is also the capital of the Central Region. Cape Coast metropolitan hospital serves an average population of 140,000 and receives a yearly attendance of 80,000 to 100,000 patients. It is located in Bakaano a suburb of Cape Coast. Elmina is the district capital of Komenda-Edina-Eguafo-Abirem (KEEA) and covers an area of 1,372.45 square kilometres. The Elmina Urban Health Centre caters for inhabitants of an area of 600 sq. km comprising 22 villages with catchment population of about 50,000. Twifo Praso is the district capital of the Twifo-Heman-Lower Denkyira district. The district covers an area of 1,199 square km with about 1,510 settlements. Twifo Praso Government hospital is a district hospital. It a serves catchment population of 15,351, distributed in Twifo Praso and neighbouring towns. The Assin North district covers an area of about 1,500 sq. km. and comprises about 1,000 settlements. The St. Francis Xavier Hospital still remains the District Hospital for both Assin north and Assin south districts covering a land mass of 1,255 sq. km (1/4) of Central Region. The hospital has catchment population of 207,000.

### Ethical considerations

The study was approved by the Ghana Health Service Ethics Committee (GHS-ERC-17/01/12) and University of Cape Coast Institutional review Board (UCCIRB/28/09/3.1.1). Approval was also sought from the Medical directors and administrators of the various health facilities before sample collection. Anonymity of study subjects was strictly enforced. Samples were collected and handled solely by trained laboratory technologists. The study posed no risk to participants except for the transient pain they felt during blood collection. Sterile techniques were employed using disposable new materials at all times.

### Recruitment of participants

The study was explained to the prospective participants who were referred to the laboratory for malaria test. Informed consent was sought from those who satisfied the inclusion criteria and agreed to be part of the study. A patient was included into the study if he/she was older than six months old, had *Plasmodium* parasitaemia, was conscious, non-haemophilic, and did not experience palpitation at the time of sample collection.

### Sample collection

One millilitre (1 ml) of blood sample was collected from each participant into tubes containing EDTA by trained and licensed medical laboratory technologists from the health facilities. Also 10 ml mid stream urine samples were collected at the time of participant visit for CQ analysis. All samples collected were stored on ice and transported to the Parasitology Research Laboratory of the Department of Biomedical and Forensic Sciences, University of Cape Coast where they were stored at-20°C. About six drops of blood sample collected was spotted on a 3MM Whatman filter paper. The blood spots were air-dried and stored at -20°C in zip-locked plastic envelopes containing silica gel.

### Detection of *pfcrt* polymorphisms

DNA was extracted from the perforated portions of blood spotted filter-paper using chelex extraction method [13]. PCR followed by restriction fragment length polymorphism (RFLP) was used to detect the mutations following published protocols [14]. The primers for the DNA amplification of *pfcrt* are shown in Table 1.

**Table 1:** **Oligonucleotide sequence and PCR conditions used to detect *Pfcrt*** **K76T SNP**

**Primer**	**Sequence (5' - 3')**	**Size (bp)**	**PCR condition**
Primary amplification			
CRT1F	TTGTCGACCTTAACAGATGGCTCAC	526	96°C, 15’ followed by 45X (96°C, 30”; 56°C, 90”; 72°C, 90”); 72°C, 10’
CRT1R	AATTTCCCTTTTTATTTCCAAATAAGGA		
Nested amplification			
CRT2F	CTTGTCTTGGTAAATGTGCTC	200	96°C, 15’ followed by 45X (96°C, 30”; 50°C, 90”; 72°C, 90”); 72°C, 10’
CRT2R	GAACATAATCATACAAATAAAGT		

### Mystery buying of CQ

Mystery buyers were used to investigate if Chemical shops and Community Pharmacies in these communities still stocked CQ using convenient sampling method. Surrogate buyers visited chemical and pharmacy shops located in the study areas to buy CQ.

### Urine CQ assay

The specific gravity of urine samples were determined using urinometer (BD Adams ^TH^, Canada). The presence of CQ in the participants’ urine was detected with Saker-Solomon’s method [15]. A standard curve was estimated from standard concentrations (0, 2, 3, 4, or 5 μg/ml) of CQ (Sigma-Aldrich, UK). The presence of CQ in the urine samples that tested positive to Saker-Solomon method was determined spectrophotometrically. In the Saker-Solomon method the urine samples were diluted and 10 μl of each sample was added to a well in triplicates. The absorbance of the urine samples was read at 565 nm. The concentrations were determined from the standard curve. A standard CQ concentration which corresponds to each of the urine samples was also prepared read at 565 nm. The cross-reactivity for each sample was then determined using the formula; Absorbance of Urine sample/Absorbance CQ standard. The urine samples that tested positive qualitatively to Saker-Solomon’s urine CQ method but the samples whose error margins fell below or above 5% error margin were considered to be cross-reacting with other compounds (such quinine) after estimating the ratios of absorbance of samples to absorbance of standards of equal concentrations.

### Data analysis

Data were entered and validated using Microsoft Office Excell® 2007 (Microsoft Corporation) and analysed with SPSS® Statistical Software version 16 (SPSS Inc.) and MedCalc® statistical software version 12.7.2 (MedCalc software, Ostend, Belgium). The count of samples with mutant and wild-type alleles was used to generate the prevalence of the alleles. Pearson chi-square test and relative risk were used to compare the risks of becoming infected with CQ-resistant *P. falciparum* when one stays in an area where pharmacies and chemical shops stock CQ. Pearson chi-square test and Odd ratio were also used to compare the risks of the use CQ and becoming infected with CQ-resistant *P. falciparum* strains. The Pearson chi-square test was used to determine the association between the alleles and CQ stock and CQ usage.

## Results

A total of 444 participants had their urine successfully examined for the presence of CQ in this study; 56 from Cape Coast, 105 from Elmina, 143 from Assin Foso and 140 from Twifo Praso. The corresponding prevalence of patients with detectable CQ in their urine per the study sites were 23.21%, 11.43%, 18.88% and 16.43% respectively. In all, 75 of participants had CQ in their urine representing 16.89% prevalence of CQ consumption in the four study areas. The total prevalence of CQ stocks within the four study areas were 14.49% (10/69); 21.05% (4/19) in Cape Coast, 9.09% (1/13) in Elmina, 16.67% (3/19) in Assin Foso and 10.53 (2/19) in Twifo Praso (Table 2).

**Table 2:** Prevalence of subjects that still use CQ and the drug shops that had CQ stocks at the study sites

**Study sites**	**Percentage prevalence of pfcrt mutations**	**Percentage of subjects with CQ in urine (n/N)**	**Percentage of drug shops with CQ stocks (n/N)**
Cape Coast	80.4	23.21 (13/56)	21.05 (4/19)
Elmina	78.8	11.43 (12/105)	9.09 (1/13)
Assin Foso	75.9	18.88 (27/143)	16.67 (3/19)
Twifo Praso	50.0	16.43 (23/140)	10.53 (2/19)
Total number	71.9	16.89 (75/444)	14.49 (10/69)

n represent the number of subjects that had CQ in their urine.

N represent the total number of subject whose urine were tested for CQ.

**Figure 1:**
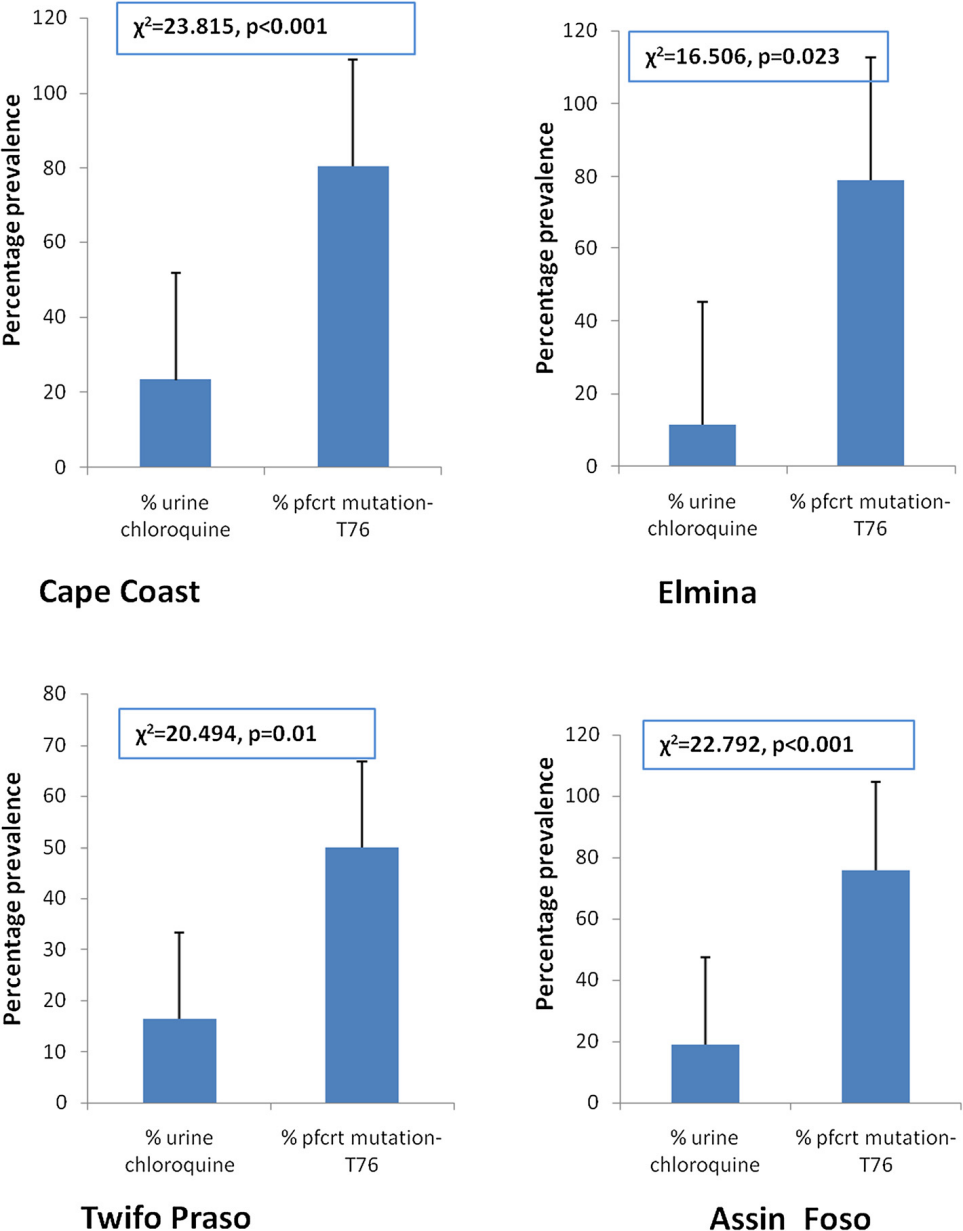
**Association between chloroquine stocking in a community and prevalence of K76T of *pfcrt *K76T mutation.** The % of community pharmacies and chemical shops that are still stocking and selling chloroquine in the study communities.

**Figure 2:**
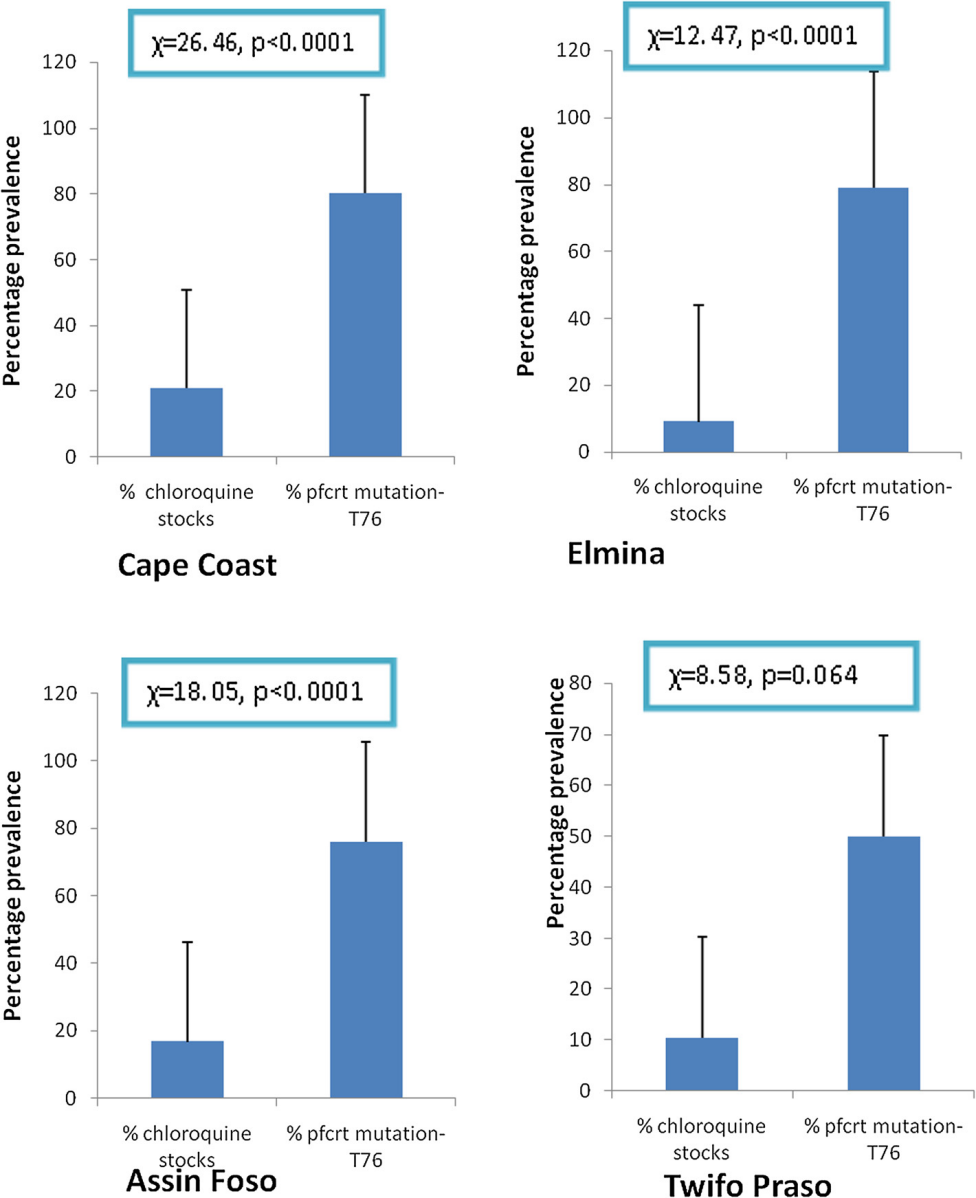
**Association between urine chloroquine levels and prevalence of K76T of *pfcrt *K76T mutation in the study sites.** The % urine chloroquine indicates the prevalence of chloroquine use among the study subjects.

Two hundred and fourteen (214) of the *P. falciparum* isolates had DNA extracted and analyzed for *pfcrt* T76 mutant allele. Fifty six (56) of the isolates were from Cape Coast, 52 from Elmina, 54 from Assin Foso and 52 from Twifo Praso. The overall prevalence of *pfcrt* T76 mutation was 71.96% (154/214); 80.4% in Cape Coast, 78.8% in Elmina, 75.9% in Assin Foso and 50.0% in Twifo Praso. The distribution of the two alleles; wild type (K76) and mutant type (T76) from the four study sites is shown in Table. Significant association was found between prevalence of CQ stocks and prevalence of *pfcrt* T76 mutant allele in all the study sites, except Twifo Praso as shown in Figure 1. Additionally, the levels of CQ usage within a study site were significantly associated with the prevalence of *pfcrt* T76 mutant allele. In Cape Coast and Assin Foso, a high number of participants (23.21 and 18.88% respectively) had CQ in their urine samples, and higher prevalence of *pfcrt* T76 mutant *P. falciparum* in their samples (p < 0.0001) as compared to Elmina (p = 0.023) and Twifo Praso (p = 0.01) as shown in Figure 2.

The study also demonstrated that individuals who stay in an area where community pharmacies and chemical shops had CQ stocks have five times more risk of become infected with T76 resistant *P. falciparum* strain than individuals who stay in CQ free area [RR = 0.20, 95% CI (0.11-0.34), p < 0.001]. As shown in Figure 1, there was significant association between CQ stock within the communities and T76 mutation except Twifo Praso. Cape Coast had the highest number of community pharmacies and chemical shops that stocked CQ and the risk of individuals becoming infected with T76 resistant mutant is 26% [RR = 0.26, 95% CI (0.11-0.63), p < 0.001] while individuals staying in Elmina, study site with the least number of community pharmacies and chemical shops stocking CQ had 10% risks of becoming infected with T76 resistant mutant [RR = 0.10, 95% CI (0.02-0.66), p < 0.05]. Again, individuals who still use CQ for malaria treatment had thirteen times more risks of harbouring T76 resistant mutant *P. falciparum* strain compared to individuals who do not use CQ [OR = 0.08, 95% CI (0.05-0.12), p < 0.001]. The risk of stocking CQ in an area or using CQ and becoming infected with *P. falciparum* with T76 mutation is shown in Table 3. CQ use was also observed to be significantly associated with T76 mutation in all the study sites (Figure 2).

**Table 3:** **Association between CQ stocks and/or CQ in urine and the risk of acquiring infection with CQ resistant *P. falciparum *** **strains**

**Study sites**	**CQ stocks and prevalence of T76 mutation of *pfcrt***		**Urine CQ and prevalence of T76 mutation of *pfcrt***	
	**RR**	**95% CI**	**OR**	**95% CI**
Cape Coast	0.26	(0.11-0.63)^HS^	0.07	(0.03-0.18)^HS^
Elmina	0.10	(0.02-0.66)^S^	0.04	(0.02-0.11)^HS^
Twifo Praso	0.21	(0.06-0.80)^S^	0.19	(0.09-0.39)^HS^
Assin Foso	0.22	(0.08-0.62)^HS^	0.06	(0.03-0.12)^HS^

HS = highly significant (p < 0.001).

S = significant (p < 0.05).

NS = not significant (p > 0.05).

## Discussion

Various studies since the change in malaria treatment policy in Ghana have reported different levels in prevalence of the mutant *pfcrt* T76 in *P. falciparum* isolates collected from different parts of the country [4,5,16]. CQ pressure is driven by high CQ usage in an area and it is a major determinant of selection and spread of CQ resistance genes among *P. falciparum* population [17-19]. The persistent treatment of *P. falciparum* infections with CQ will subsequently encourage the selection of CQ resistant strains [20-23]. The danger is that there could be subsequent selection of additional changes in genes regulating *P. falciparum* response to CQ [24-28]. Such a situation will favour cross-resistance between CQ and amodiaquine, which is co-partner of artesunate, the first ACT deployed in Ghana [28-30].

In this study, the risks of becoming infected with T76 mutant of *pfcrt* gene of *P. falciparum* isolates was strongly associated with staying at a place where community pharmacies and chemical shops stock CQ for sale or where there is CQ usage. This observation is supported by several field studies in different geographical sites that have shown absolute specificity of *pfcrt* T76 molecular marker to predict CQ resistance both *in vitro* and *in vivo*[31,32]*.* The high prevalence of the *pfcrt* T76 mutation recorded in this study after eight years of discontinuous usage of CQ in Ghana is an indication of circulation of CQ resistant strains. The transmission of the T76 mutant of *pfcrt* gene is possible in the study areas because it has been shown elsewhere that there is a high gametocyte carriage and infectivity of resistant parasites by mosquitoes than the drug sensitive types [33,34]. The detection of traces of CQ in the urine samples of study subjects confirms CQ usage, a factor responsible for the development of CQ resistance. It is, therefore, speculated that CQ usage and not AS-AQ combination usage that selects *pfcrt* T76 resistance. The observed high prevalence of the pfcrt T76 mutation is similar to the Uganda study where CQ was substituted with artemether-lumefantrine (AL) as the first-line anti-malarial drug [35,36]. After seven years of AL usage, CQ resistance was still high, ranging from 98.7 to 100% [35,36]. The increase in the observed resistance in those reports was attributed to continuous use of CQ, the same reason adduced by this study. In support of this speculation are two separate reports that did not find any association between pfcrt T76 and sulphadoxine-pyrimethamine-amodiaquine combination in treatment failure after four years [37] as well as pfcrt T76 *in vitro* resistance studies after eight years of using amodiaquine combination at Pikine, Senegal [38].

It has been demonstrated in this study that the level of CQ stocking and usage is significantly associated with the prevalence of *pfcrt* T76 mutation within a study site. This support the argument that emergence of CQ resistance is influenced by intensity of CQ usage [39-42]. The mutations in *pfcrt* have been identified to confer and improve the fitness and stability of parasites containing the T76 mutation [43,44]. The detection of high prevalence of some point mutations of *pfmdr1* in Central region of the country (unpublished data) also augments the fitness and stability of CQ resistant *P. falciparum* strains in the study sites. The current high prevalence of *pfcrt* threatens anti-malarial treatment policy in the country. Since mutations in *pfcrt* have shown to confer resistance to amodiaquine which co-partners artesunate in the combination therapy as first line malaria treatment regimen in the country, it is important to constantly monitor the efficacy of all ACT regimen especially amodiaquine-based ACT for early detection of *P. falciparum* resistance [16,26,32,45-48]. Also the level of quinine and amodiaquine resistance [5] as well as treatment failures with artesunate + amodiaquine in the country requires constant monitoring.

The differences in the distribution of *pfcrt* T76 resistance gene among the four study sites were constantly related to the different pattern of CQ stocking and usage in each area. This suggests that complete withdrawal of CQ from the country could lead to re-emergences of CQ sensitive *P. falciparum* similar to the findings from other endemic countries [35,36,49,50].

The reason for stocking CQ by community pharmacies and chemical shops was unknown, however CQ is cheaper and this may account for people patronizing [51,52] it after its ban. Additionally, subjects who have strong perceptions about the efficacy and efficiency of CQ for malaria treatment [51-53] still patronized it. Therefore, switching from CQ to ACTs has become very difficult for them. Other studies conducted in various parts of Ghana and other countries also indicated the continuous usage of CQ [53-55].

## Conclusion

The findings gave evidence that the significant variations in *pfcrt* T76 of *P. falciparum* isolates in some parts of Ghana are highly associated with the sale and use of CQ in these communities.

## Competing interests

The authors declared that there is no conflict of interest.

## Authors’ contributions

KKA performed the experiment experiments, interpreted the data and drafted the manuscript. KKA, JNB, NBQ, RS designed the study. JNB, NBQ supervised the entire work and revised the manuscript. KKA, RA, EOA participated in laboratory analysis, provided statistical expertise in analysing the data and participated in drafting the manuscript. All authors read and approved the final version of the manuscript.
